# Supplementary material for: Dependence on Myb expression is attenuated in myeloid leukaemia with N-terminal CEBPA mutations
Source: Life Sci Alliance. 2019 Mar 15;2(2):e201800207. doi: 10.26508/lsa.201800207 (PMC6421631; doi:10.26508/lsa.201800207)
Supplement: Supplementary file 1 [file LSA-2018-00207_Supplemental_Information_1.docx]

**Supplementary information**

**Dependence on *Myb* expression is attenuated in myeloid leukaemia with N-terminal CEBPA mutations**

**Volpe G. et al**

**RNA sequencing**

Reads were aligned to the mm9 genome using tophat 2.0.14 (Trapnell et al., 2009), with -G mm9_refFlat.gtf as the transcript annotation parameter and --library-type fr-firststrand as the library preparation protocol parameter. Biological replicates were aligned independently and technical replicates were subsequently merged using samtools merge 1.3.1 (Li et al., 2009). For read coverages only, biological replicates were merged using samtools merge and resulting bam files using were used as input for bedtools genomecov 2.19.0 (Quinlan and Hall, 2010) with -ibam -bg -split -trackline -trackopts as parameters, to obtain concise graphical output. Differential gene expression analysis was performed using cuffdiff 2.2.1 (Trapnell et al., 2012) using mm9_refFlat.gtf as the supplied transcript annotation and --library-type fr-firststrand as the library preparation protocol parameter but otherwise default parameters**,** and a coma-separated list of bam files reflecting individual replicates for each condition as input. Differentially expressed genes were filtered based on the significant flag output by cuffdiff (i.e. based on FDR q-value <0.05). Venn diagram overlaps were performed using BioVenn (Hulsen et al., 2008). Generation of fragments per kilobase of transcript per million mapped reads (FPKM) counts for all experiments and normalisation was performed using cuffnorm 2.2.1 on the mm9_refFlat.gtf transcript annotation, using --library-type fr-firststrand as the library preparation protocol parameter, with otherwise default parameters. Steady-state Spearman correlation clustering was performed in R using method=”spearman” as a parameter, with pdf output generated with the heatmap.2 function of the R gplots package (RDevelopment CORE TEAM, 2008; Warnes, 2006). To avoid log_2_ transform of 0 values for transcripts showing no expression, log2 FPKMs were treated as log_2_(FPKM+1). Hierarchical clustering of log_2_ fold changes was performed using cluster 3.0(de Hoon et al., 2004) using -e 5 -g 5 (Spearman correlation as the distance metric for gene and array clustering) and -m m (complete linkage) as parameters, following log_2_ transform and subtraction. Heatmap images were generated via Java TreeView (Saldanha, 2004).

**ChIP-Seq data processing and analysis**

Reads were aligned to the mm9 genome using bowtie 0.2.12 (Langmead et al., 2009), using -v 2 --best --strata --tryhard -m 1 as parameters. Peak detection and coverage track generation were carried out using macs2 callpeak 2.1.0 (Zhang et al., 2008) using --keep-dup -g mm --B --trackline --SPMR. Log_2_ fold change ranking of C/EBPα p30/p42 was essentially adapted from a previously described method (Cauchy et al., 2016), whereby summit files were first concatenated using the bash cat command, then subsequently sorted via bedtools sort, merged using bedtools merge -d 200. The resulting bed file corresponding to merged summits was used to retrieve tag counts ± 1 kb via Homer annotatePeaks (Heinz et al., 2010) using -hist -ghist 10 -bedGraph -size 2000 as parameters. Resulting matrices were sorted on log_2_ tag count fold change ± 200 bp, with sorted bed file output subsequently used to retrieve tag counts from other experiments. Matrices were imaged using Java TreeView. Specific groups were defined as follows with respect to log_2_ p30/p42 tag count fold change (FC): p42-specific, FC≤-1; shared, -1<FC<1; p30-specific, FC ≥1.

**Published ChIP-Seq data processing**

Fastq files were generated on-the-fly via the sra-dump command of the sratoolkit 2.8.0 package (Leinonen et al., 2011) using the -A switch to directly access online datasets for Myb (SRR1811502, SRR1811503), Cebpa (SRR1811498) and Cebpb (SRR1811501) ChIP-Seq data in RN2 (MLL-AF9/NrasG12D) murine AML cell line (Roe et al., 2015); as well as for Ets1 (SRR574737, SRR574738), JunD (SRR478496, SRR478497), p300 (SRR478455, SRR478456), CTCF (SRR478460, SRR478461), Nrf2 (SRR574764, SRR5747645) and Sin3A (SRR574742, SRR574743) ChIP-Seq data in MEL cells (Yue et al., 2014), piping the output to stdout using the -Z switch to bowtie2 2.2.8 to the mm9 genome, using --very-sensitive-local as a parameter. ChIP-Seq data from Grebien et al (Grebien et al., 2015). were obtained as bam files aligned to mm9. Peak detection and coverage track generation were carried out using macs2 callpeak 2.1.0 using --keep-dup -g mm --B --trackline --SPMR.”

.

**Published gene expression analysis**

Expression microarray data from Grebien et al (Grebien et al., 2015) were processed using the Affymetrix Expression Console software, using RMA normalisation outputting log_2_ intensity values. Gene annotation was performed using the MoGene 1.0 ST na33.2 mm9 transcript annotation. Expression data were further aggregated by gene using the aggregate function of R and further normalised using the normalizeQuantiles function of the limma package (Smyth, 2005).

**Motif discovery and heatmaps**

Motif discovery was carried out via Homer findMotifsGenome using default parameters (200 bp search region). Motif matrices were obtained via Homer annotatePeaks using -m -size 2000 on log_2_ tag count fold change sorted bed files and imaged using Java TreeView.

**Gene ontology analyses**

For gene ontology analyses entailing gene expression data, ontology signatures were retrieved from the gene set enrichment analysis (GSEA) online repository (Subramanian et al., 2005) using gene sets defined in Table S1. Matrices reflecting the FPKMs for each ontology class were obtained using the merge function of R. Average log_2_ fold changes for all classes were concatenated into one object to reflect global log_2_ gene expression fold changes per ontology class. Clustering was performed using cluster 3.0, with images generated using Java TreeView. For gene ontology analyses in ChIP-Seq data, the gene ontology function of Homer annotatePeaks was employed using the -go switch.

**Gene set enrichment analysis**

For gene set enrichment analysis (GSEA) (Subramanian et al., 2005) involving RNA-Seq data (FMH9, KL, and LL), in order to avoid log_2_ transform of 0 values and to thus correctly use the difference of classes ranking metric of GSEA, log_2_ FPKM values +1 were written to a GSEA expression file for all replicates for all conditions. For GSEAs involving expression microarray data (FDC-P1), log_2_ intensities were used in a GSEA expression encompassing all such data. A phenotype cls file was generated to reflect expression groups. To obtain gene names for C/EBPα binding sites, bedtools closest was used using -a <peak file> -b mm9_refFlat.bed -t first. For GSEA analysis, computationally optimal for gene sets below 1000 names, the top 1000 p30 and p42 peaks were selected based on summit height. GSEA was run using the above-mentioned datasets, using the GENE_SYMBOL chip annotation for the gene set, collapse dataset to gene symbols set to false, gene set permutation, difference of classes as the ranking metric, and weighted enrichment statistic.

**References**

Cauchy, P., Maqbool, M.A., Zacarias-Cabeza, J., Vanhille, L., Koch, F., Fenouil, R., Gut, M., Gut, I., Santana, M.A., Griffon, A.*, et al.* (2016). Dynamic recruitment of Ets1 to both nucleosome-occupied and -depleted enhancer regions mediates a transcriptional program switch during early T-cell differentiation. Nucleic Acids Res *44*, 3567-3585.

de Hoon, M.J., Imoto, S., Nolan, J., and Miyano, S. (2004). Open source clustering software. Bioinformatics *20*, 1453-1454.

Grebien, F., Vedadi, M., Getlik, M., Giambruno, R., Grover, A., Avellino, R., Skucha, A., Vittori, S., Kuznetsova, E., Smil, D.*, et al.* (2015). Pharmacological targeting of the Wdr5-MLL interaction in C/EBPalpha N-terminal leukemia. Nat Chem Biol *11*, 571-578.

Heinz, S., Benner, C., Spann, N., Bertolino, E., Lin, Y.C., Laslo, P., Cheng, J.X., Murre, C., Singh, H., and Glass, C.K. (2010). Simple combinations of lineage-determining transcription factors prime cis-regulatory elements required for macrophage and B cell identities. Mol Cell *38*, 576-589.

Hulsen, T., de Vlieg, J., and Alkema, W. (2008). BioVenn - a web application for the comparison and visualization of biological lists using area-proportional Venn diagrams. BMC Genomics *9*, 488.

Langmead, B., Trapnell, C., Pop, M., and Salzberg, S.L. (2009). Ultrafast and memory-efficient alignment of short DNA sequences to the human genome. Genome Biol *10*, R25.

Leinonen, R., Sugawara, H., Shumway, M., and International Nucleotide Sequence Database, C. (2011). The sequence read archive. Nucleic Acids Res *39*, D19-21.

Li, H., Handsaker, B., Wysoker, A., Fennell, T., Ruan, J., Homer, N., Marth, G., Abecasis, G., Durbin, R., and Genome Project Data Processing, S. (2009). The Sequence Alignment/Map format and SAMtools. Bioinformatics *25*, 2078-2079.

Quinlan, A.R., and Hall, I.M. (2010). BEDTools: a flexible suite of utilities for comparing genomic features. Bioinformatics *26*, 841-842.

RDevelopment CORE TEAM, R. (2008). R: A language and environment for statistical computing (R foundation for statistical computing Vienna, Austria).

Roe, J.S., Mercan, F., Rivera, K., Pappin, D.J., and Vakoc, C.R. (2015). BET Bromodomain Inhibition Suppresses the Function of Hematopoietic Transcription Factors in Acute Myeloid Leukemia. Mol Cell *58*, 1028-1039.

Saldanha, A.J. (2004). Java Treeview--extensible visualization of microarray data. Bioinformatics *20*, 3246-3248.

Smyth, G.K. (2005). limma: Linear Models for Microarray Data. In Bioinformatics and Computational Biology Solutions Using R and Bioconductor, R. Gentleman, V.J. Carey, W. Huber, R.A. Irizarry, and S. Dudoit, eds. (New York, NY: Springer New York), pp. 397-420.

Subramanian, A., Tamayo, P., Mootha, V.K., Mukherjee, S., Ebert, B.L., Gillette, M.A., Paulovich, A., Pomeroy, S.L., Golub, T.R., Lander, E.S.*, et al.* (2005). Gene set enrichment analysis: a knowledge-based approach for interpreting genome-wide expression profiles. Proc Natl Acad Sci U S A *102*, 15545-15550.

Trapnell, C., Pachter, L., and Salzberg, S.L. (2009). TopHat: discovering splice junctions with RNA-Seq. Bioinformatics *25*, 1105-1111.

Trapnell, C., Roberts, A., Goff, L., Pertea, G., Kim, D., Kelley, D.R., Pimentel, H., Salzberg, S.L., Rinn, J.L., and Pachter, L. (2012). Differential gene and transcript expression analysis of RNA-seq experiments with TopHat and Cufflinks. Nat Protoc *7*, 562-578.

Warnes, G. (2006). gplots: Various R programming tools for plotting data. Included R source code and/or documentation contributed by Ben Bolker and Thomas Lumley. R package version 23.

Yue, F., Cheng, Y., Breschi, A., Vierstra, J., Wu, W., Ryba, T., Sandstrom, R., Ma, Z., Davis, C., Pope, B.D.*, et al.* (2014). A comparative encyclopedia of DNA elements in the mouse genome. Nature *515*, 355-364.

Zhang, Y., Liu, T., Meyer, C.A., Eeckhoute, J., Johnson, D.S., Bernstein, B.E., Nusbaum, C., Myers, R.M., Brown, M., Li, W.*, et al.* (2008). Model-based analysis of ChIP-Seq (MACS). Genome Biol *9*, R137.
