# Supplementary material for: Dependence on Myb expression is attenuated in myeloid leukaemia with N-terminal CEBPA mutations
Source: Life Sci Alliance. 2019 Mar 15;2(2):e201800207. doi: 10.26508/lsa.201800207 (PMC6421631; doi:10.26508/lsa.201800207)
Supplement: Supplementary file 2 [file LSA-2018-00207_TableS1.docx]

| Gene sets used in this study: |
| --- |
| GO_POSITIVE_REGULATION_OF_EXECUTION_PHASE_OF_APOPTOSIS |
| GAL_LEUKEMIC_STEM_CELL_UP |
| GAL_LEUKEMIC_STEM_CELL_DN |
| BOGNI_TREATMENT_RELATED_MYELOID_LEUKEMIA_UP |
| BOGNI_TREATMENT_RELATED_MYELOID_LEUKEMIA_DN |
| GSE15330_LYMPHOID_MULTIPOTENT_VS_GRANULOCYTE_MONOCYTE_PROGENITOR_DN |
| GO_NEGATIVE_REGULATION_OF_CELL_CYCLE_G1_S_PHASE_TRANSITION |

**Table 1**: Gene sets used for gene ontology analyses in this study
